# Disulfide Bridges Remain Intact while Native Insulin Converts into Amyloid Fibrils

**DOI:** 10.1371/journal.pone.0036989

**Published:** 2012-06-01

**Authors:** Dmitry Kurouski, Jacqueline Washington, Mehmet Ozbil, Rajeev Prabhakar, Alexander Shekhtman, Igor K. Lednev

**Affiliations:** 1 Department of Chemistry, University at Albany, State University of New York, Albany, New York, United States of America; 2 Department of Chemistry, University of Miami, Coral Gables, Florida, United States of America; Consiglio Nazionale delle Ricerche, Italy

## Abstract

Amyloid fibrils are β-sheet-rich protein aggregates commonly found in the organs and tissues of patients with various amyloid-associated diseases. Understanding the structural organization of amyloid fibrils can be beneficial for the search of drugs to successfully treat diseases associated with protein misfolding. The structure of insulin fibrils was characterized by deep ultraviolet resonance Raman (DUVRR) and Nuclear Magnetic Resonance (NMR) spectroscopy combined with hydrogen-deuterium exchange. The compositions of the fibril core and unordered parts were determined at single amino acid residue resolution. All three disulfide bonds of native insulin remained intact during the aggregation process, withstanding scrambling. Three out of four tyrosine residues were packed into the fibril core, and another aromatic amino acid, phenylalanine, was located in the unordered parts of insulin fibrils. In addition, using all-atom MD simulations, the disulfide bonds were confirmed to remain intact in the insulin dimer, which mimics the fibrillar form of insulin.

## Introduction

Protein aggregates play an important role in living cells due to their ubiquity. Aggregation of proteins results in the formation of long, unbranched β-sheet-rich structures, commonly known as amyloid fibrils [1]. These fibrils are found as deposits in the tissues and organs of patients with various amyloid-associated diseases, such as Alzheimer’s disease (AD), Parkinson’s disease (PD), Huntington’s disease (HD), prion disease, and type II diabetes [2], [3]. There is also increasing evidence that small aggregates of misfolded proteins are most toxic and the formation of amyloid fibrils is a defense mechanism [4]. It is known that more than 20 proteins that can aggregate to form amyloid-like fibrils. Previously, it was proposed that the ability to form amyloid fibrils is not a peculiarity of this small group of disease-related proteins, but rather, the ability to form amyloids is a generic property of the polypeptide chain [5]. Thus, many physiochemical properties of protein sequences, such as charge, hydrophobicity, and the tendency to form secondary structures, were extensively elucidated in recent decades to understand their relative propensities for amyloid fibril formation. One example of these properties is disulfide bonds, which are present in 65% of all secreted proteins, and in 50% of proteins involved in amyloidosis [6].

The behavior of disulfide bonds upon protein aggregation has been extensively studied over the past decade [7], [8], [9]. Disulfide bonds limit the way in which a protein or a peptide can aggregate into a fibril via steric restraint. For example, the reduction of intra-molecular disulfide bonds in β_2_ microglobulin was determined to limit the formation of long fibrils upon protein aggregation [9], [10]. In our previous work, we demonstrated that a reduction of three out of four disulfide bonds in bovine apo-α-lactalbumin leads to significant changes in the aggregation pathways of these proteins, as well as the structure and morphology of their mature fibrils [11].

There is great interest in understanding the influence of disulfide bonds on the stability of insulin. The polypeptide hormone insulin stimulates a complex signal transduction pathway associated with glucose metabolism. The native structure of the insulin monomer is mainly helical, with two of its polypeptide chains linked by one intra-chain and two inter-chain disulfide bonds. Importantly, disulfide bonds are critical for the physiological function of insulin [12]. Insulinoma and injection amyloidosis are associated with insulin aggregation [13], [14]. Zako *et al.* showed that reducing all disulfide bonds of native insulin leads to the formation of structurally and morphologically different insulin fibrils [15]. In addition to the dramatic impact on insulin stability and aggregation, disulfide bonds can contribute to free radical formation and fibrillar toxicity. In particular, Schöneich proposed that sulfur-containing amino acids cause free radical shrapnel during protein aggregation [16]. However, whether disulfide bonds undergo cross-scrambling during insulin aggregation, their role in this process, and their location in the fibrillar structure remain unknown.

Insulin is present as a dimer in solution. However, only the insulin monomer is physiologically active [17]. Insulin dimerization has been proposed as a key step in the amyloidogenic pathway [18]. Belfort *et al.* proposed that three dimers of insulin comprise the fibril precursors that function as a template for further insulin aggregation [19]. Insulin fibrils are β-sheet-rich aggregates, whereas native insulin has a predominantly α-helical structure. Thus, an extensive α-helical to β-sheet refolding should occur during the fibrillation process. The elucidation of the amyloidogenesis of the insulin sequence, which is a primary determinant in protein aggregation, has been a topic of active research in recent decades [20], [21], [22]. New possibilities could be created for specific drug design to block insulin aggregation and fibril formation. Eisenberg *et al.* proposed that part of the B-chain sequence, LVEALYL, is the smallest segment responsible for the initiation of insulin aggregation [18]. However, this segment has also been determined to terminate protein aggregation. Previous studies by Sawaya *et al*. have demonstrated that several other sequences, such as LYQLEN (residues A13–A18) and VEALYL (residues B12–B17), also modify protein aggregation and form amyloid fibrils [23], [24]. In addition, point mutations have been found to either delay or prolong the lag phase of insulin fibrillation [21], [22]. However, whether the studied amino acid fragment is located in parts of the unordered fibril or forms the core spine remains elusive.

Hydrogen-deuterium (H/D) exchange is a valuable tool for characterizing protein structure, solvation, and water exposure when combined with NMR, mass spectrometry, and vibrational spectroscopy. Coupling NMR with H/D exchange has been demonstrated to be a powerful method for determining the amino acid motif involved in β_2_ microglobulin fibril formation [25]. Deep UV resonance Raman spectroscopy combined with H/D has also been shown to be a very powerful tool for fibril core characterization [26], [27]. In an amino acid residue, the main chain NH group and O-, N-, and S-bound protons exchange easily, whereas carbon-bound hydrogens do not. In the hydrophobic core or strongly hydrogen-bonded secondary structures of proteins, the H/D exchange rates are strongly reduced due to the shielding of exchangeable sites. Previously, the hydrophobic fibril core was demonstrated to be highly resistant to H/D exchange [27]. The current model of amyloid fibrils postulates that a highly hydrophobic cross-β core is flanked by unordered parts. Taking this model into account, one can expect that hydrogen-deuterium exchange of these fibril structures will result in the proton exchange only in unordered parts, whereas the cross-β core remains protonated.

Herein, using the combination of deep ultraviolet resonance Raman (DUVRR) and Nuclear Magnetic Resonance (NMR) spectroscopy with H/D exchange, we determined the parts of the insulin sequence that form the fibril core and are present in the unordered parts. We observed that at least two B-chain segments, B3–B7 and B10–B18, remain highly protonated under H/D exchange and most likely form the fibril core. Surprisingly, we did not observe any highly protonated segments in the insulin A-chain that were longer than two amino acid residues. We also found that one cysteine residue of each disulfide pair is located in the hydrophobic fibril core, whereas the other residue sticks out of the fibril core and is most likely located in the unordered parts of the fibril. This discovery demonstrates that fibril disulfide bonds remain intact with the same molecular conformation as in native insulin, even after an extensive conversion of the α-helical structure to a fibrillar β-sheet. One can envision that during protein aggregation, cysteine disulfide bonds extend out into the aqueous media, whereas tyrosines are packed inside the fibril cross-β core. We performed MD simulations in aqueous solution to model the conversion of mainly α-helical monomers into primary β-sheet dimers. Our results indicate that the monomer aggregation process occurs via a zipper-like mechanism as previously proposed by Eisenberg and co-workers [18]. The complete melting of α-helices and the formation of a significant amount of β-sheets occur, although all three disulfide bonds of native insulin remain intact.

## Results and Discussion

### Determination of Amino Acid Protection

**Figure 1:**
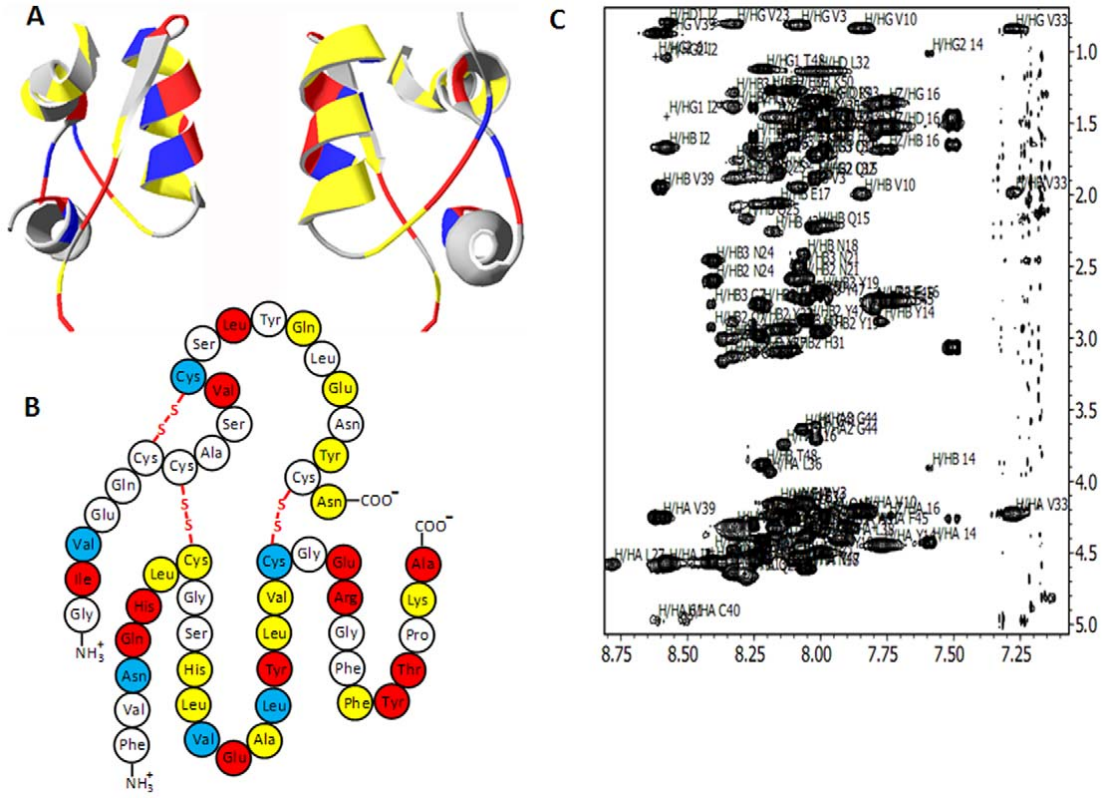
The majority of H/D exchange-protected amino acids are located in the B-chain of insulin. The amino acids in the 3D structure (A) and primary sequence of insulin fibrillar monomer (B) are colored according to the following degree of H/D exchange protection: yellow, I_D2O_/I_H2O_≥0.75; red, 0.75>I_D2O_/I_H2O_≥0.675; and blue, 0.675>I_D2O_/I_H2O_≥0.60. Gray (in the 3D structure) and white colors (in the primary sequence) indicate unprotected (exchanged) amino acids. (C) 2D ^1^H,^1^H-TOCSY spectrum of insulin fibrillar monomer in DMSO and 0.05% TFA at 30°C after a 7-day D_2_O exchange at room temperature. NMR data were acquired at room temperature on a Bruker Avance II 500 MHz NMR spectrometer equipped with a cryoprobe.

After the termination of insulin fibrillation, mature insulin fibrils, separated from un-aggregated protein, were re-dispersed in D_2_O, pD* 1.9 at 25°C. Deuterium atoms were substituted for hydrogen atoms in fibril unordered parts, while the highly hydrophobic core remained protonated. Our microscopic observation of insulin fibrils before and after H/D exchange did not show any noticeable changes in their morphology (Figure S1). The exchanged fibril solution was then lyophilized and re-dissolved in a buffer composed of 99.95% DMSO and 0.05% TFA, which disintegrates the fibril structure into protein monomers (Figure S2) without changing the protonation state of amide protons. The resulting protein was then analyzed by homonuclear NMR spectroscopy. The un-exchanged amides from the amino acids that were localized in the fibril core were detected, and the amino acid residues that exchanged a proton for a deuterium were “invisible.” To determine the degree of protection for each residue that remained protonated, the peak intensities of insulin that was incubated with and without D_2_O were plotted against the residue number (Figure 1 and Figure S3). The following three thresholds were established to illustrate the degree of protection: yellow, I_D2O_/I_H2O_ ≥0.75; red, 0.75>I_D2O_/I_H2O_≥0.675; and blue, 0.675>I_D2O_/I_H2O_≥0.60 (Figure 1a and b).

Because a native source of insulin was used, we could not isotopically enrich insulin with NMR-active nuclei, such as ^13^C and ^15^N, which help facilitate the chemical shift assignment process [28]. Therefore, 2D ^1^H,^1^H-total correlation spectroscopy (TOCSY) and nuclear Overhauser effect spectroscopy (NOESY) were used for the sequence-specific assignment of the chemical shifts of protons in the amino acid residues of the protein. ^1^H,^1^H TOCSY experiments were used to determine the types of amino acid residues that were present. ^1^H,^1^H NOESY experiments were used to place residues within the protein primary sequence for sequence-specific assignments [29].

The spectral dispersion of insulin in the amide proton region was limited, ranging from 7.1 ppm to 8.8 ppm (Figure 1c), which is a strong indication that insulin is largely devoid of tertiary structure under the NMR buffer conditions. This relatively narrow spectral region leads to the significant spectral overlap of proton resonances, complicating the chemical shift assignment. When analyzing the fibril monomer, we unambiguously assigned 35 of the 49 amino acid insulin residues resolved in 2D ^1^H,^1^H-TOCSY spectra. However, 14 residues (A chain: Cys^6^, Cys^7^, Ser^9^, Ser^12^, Tyr^14^, Leu^16^, Asn^18^, Cys^20^; B chain: Phe^22^, Gly^29^, Ser^30^, Gly^41^, Gly^44^, Phe^45^) were missing and could not be assigned due to either spectral overlap or extreme line broadening caused by the intermediate exchange between conformers in solution. It is also possible that amino acid residues located in unordered fibril parts exchanged with deuterium at rates that were too fast to be detected by NMR spectroscopy.

Only two amino acid doublets, Ile^2^-Val^3^ and Val^10^-Cys^11^, in the A-chain were observed to be protected to a medium and low extent (60–75%). In the rest of the A-chain, four single amino acids, Gln^15^, Glu^17^, Tyr^19^ and Asn^21^, were observed to have high (over 75%) protection, and Leu^13^ was observed to have medium (67.5 to 75%) protection. Interestingly, these amino acid residues alternate with the following unprotected residues, which have even numbers: Ser^12^, Tyr^14^, Leu^16^, Asn^18^ and Cys^20^. One might expect that the protected amino acid residues from the A-chain form the core spine, whereas the rest of the residues (57%) are most likely located in the unordered parts of the fibril.

In the B-chain, most amino acid residues (73%) are protected. We identified two segments [(Asn^3^, Gln^4^, His^5^, Leu^6^ and Cys^7^); (His^10^, Leu^11^, Val^12^, Glu^13^, Ala^14^, Leu^15^, Tyr^16^, Leu^17^, Val^18^ and Cys^19^ )] that remain protonated under H/D exchange. Close to the N-terminus, three amino acids (Phe^25^, Tyr^26^ and Thr^27^) along with Lys^29^ and Ala^30^ are also protonated to medium and high extents. Among the protonated amino acid residues of the B-chain, there is an LVEALYLV segment predicted by Eisenberg to be the main contributor to the fibrillar core formation [18]. We also found that the following N-termini of both chains are protected: Asn^21^ of the A-chain and Ala^30^ of the B-chain to a medium and a high extent, respectively. However, the C-termini of both chains remain unprotected, suggesting that the protein C-terminus is located outside the core, whereas the N terminus takes part in fibrillar core formation. Our calculations show that in both A- and B-chains, 61% of the insulin sequence remains protonated and located in the fibril core. We also determined that three of four tyrosine residues were protected and were most likely present in the hydrophobic fibril core. However, another aromatic amino acid, phenylalanine, remained mostly unprotected (only one of three amino acid residues is protected) in the fibril structure.

Three disulfide bridges have been shown to play a vital role in the stability of the insulin monomer. Taking into account the dramatic perturbation of the insulin secondary structure from an α-helix into a β-sheet upon protein aggregation, the stability of the disulfide bridges during this process was investigated. Prior to this study, there was no experimental evidence about the scrambling or stability of these bridges [7], [8]. Our results show that insulin fibrils have an intriguing organization of cysteine such that one of each cysteine pair is protected (Cys^11^, Cys^7^ and Cys^19^), and the other is not (Cys^6^, Cys^7^, and Cys^20^). We found that Cys^11^, Cys^7^ and Cys^19^ have a higher degree of protection of 60% for H/D exchange and are most likely located in the hydrophobic fibril core. These data suggest that each unprotected cysteine may “follow” its protected partner, which is integrated into the β-sheet core, during secondary structure changes, leaving the disulfide bond intact. Therefore, we hypothesized that the unordered parts of the fibril contain cysteine and are rich in disulfide bonds. One might speculate that the specific location of cysteine may play a role in intertwining proto-filaments and proto-fibrils. As a result, Raman spectroscopy was used to investigate the conformations of disulfide bridges in native insulin and insulin fibrils.

### The Conformations of Disulfide Bonds

**Figure 2:**
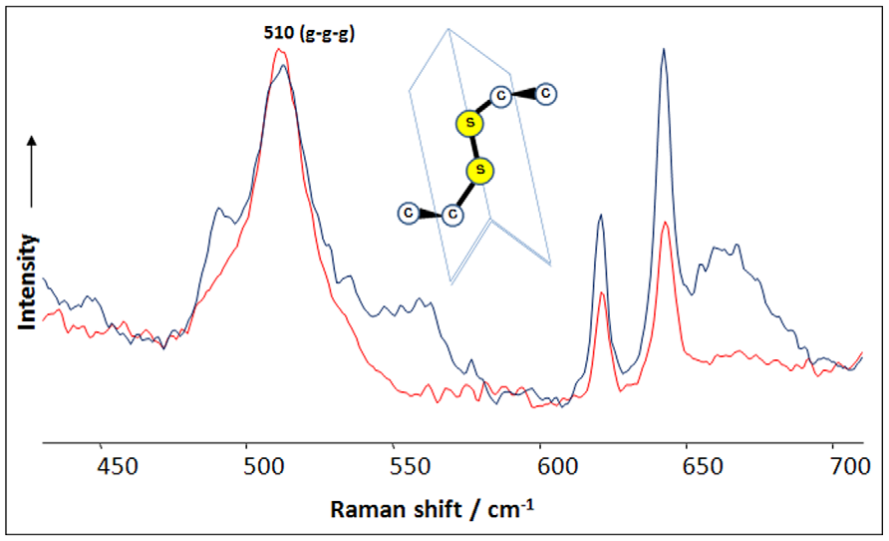
Disulfide bonds preserve their conformation upon insulin fibrillation. Raman spectra of native insulin (red) and insulin fibrils (black) have a peak at 510 cm^−1^, corresponding to the gauche-gauche-gauche (g-g-g) conformation of disulfide bonds (schematically represented in the inset).

There are three disulfide bonds that maintain the structure of the insulin monomer. Their scrambling and stability upon fibrillation have been extensively studied [7], [8], [13]. Raman spectroscopy is a unique technique used in the structural characterization of protein disulfide bond conformations. Structural information can be obtained regarding the internal rotation of C-C-S-S-C-C bonds that are present in the following conformations: gauche-gauche-gauche (g-g-g), gauche-gauche-trans (g-g-t), and trans-gauche-trans (t-g-t) [30]. Using non-resonance Raman spectroscopy with excitation at 785 nm, we determined that the predominant gauche-gauche-gauche (g-g-g) conformation for all fibril disulfide bonds is identical to that of disulfide bonds in the native protein (peak at 510 cm^−1^, Figure 2).

These data indicate that all three disulfide bonds do not break, and all three keep the predominant gauche-gauche-gauche conformation of the C-C-S-S-C-C segment. Insulin aggregates and preserves its disulfide bonds by squeezing one cysteine residue in each disulfide bond outside the fibril core to the flexible unordered parts. Based on this observation, we hypothesized that the surface of the insulin fibrils should be rich with cysteine residues and disulfide bonds, which may play a significant role in the high free radical activity that is associated with sulfur atoms [16]. Additionally, the NMR data support the conclusion that disulfide bonds do not scramble during insulin aggregation. The scrambling of disulfide bonds would lead to changes in the orientation of the protein backbone around these cysteine residues, resulting in large chemical shifts for the affected residues. We observed that the amino acid peak positions within the vicinity of cysteine residues in the 2D ^1^H,^1^H-TOCSY spectra of an insulin fibril monomer and native protein are similar, indicating that the disulfide bonds did not scramble upon protein aggregation (Figure S4). Our NMR and Raman data provide new insights about insulin fibril surface organization, which may serve as a basis for the design of therapeutic drugs.

### Secondary Structure of the Fibril Core

**Figure 3:**
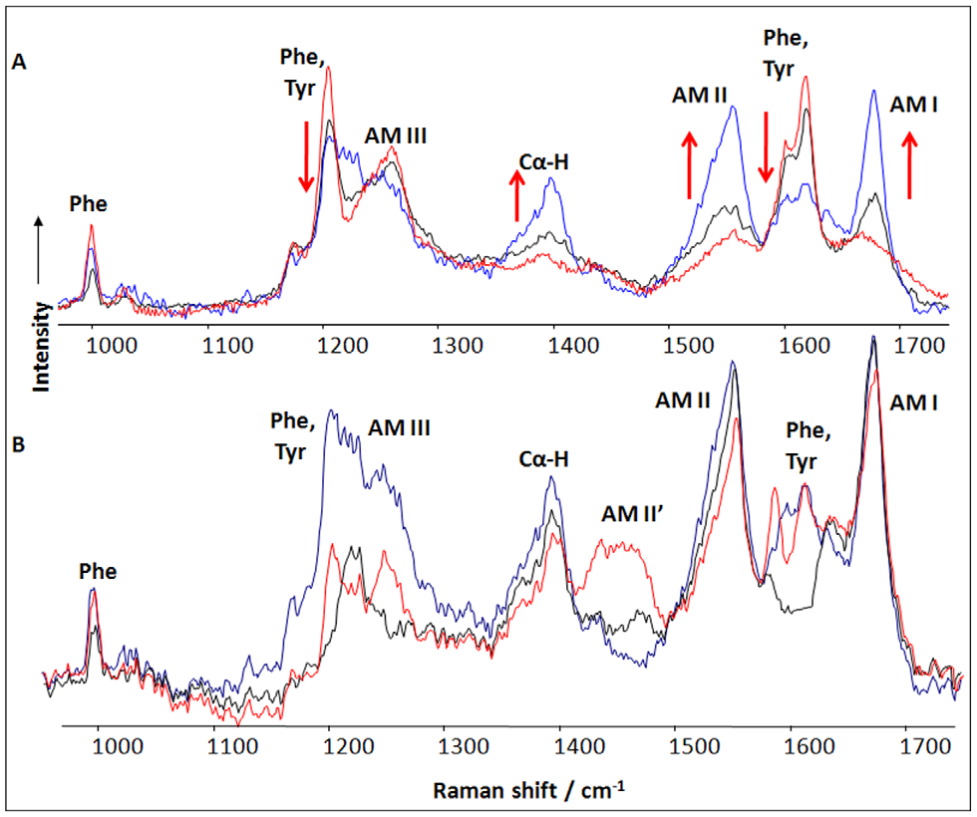
The secondary structure of insulin changes dramatically from being mostly α-helical in the native protein to highly β-sheet-rich in the fibrillar form. The local environment of tyrosine residues changes simultaneously during hydrophilic to hydrophobic protein aggregation. A) DUVRR spectra of bovine insulin at pH 2.0, 25°C (red) incubation solution after 30 min (black) and one hour (blue) of heating at 70°C. Simultaneously, approximately 75% of the insulin sequence is packed into the cross-β-core, and the remainder forms unordered parts of fibrils. B) Deep UV resonance Raman spectra of insulin fibrils in H_2_O (blue), after deuteration (red), and a spectrum of cross-β-sheets (black). The contribution of aromatic amino acids is quantitatively removed by subtracting the spectra of phenylalanine and tyrosine.

Deep UV resonance Raman spectroscopy has been demonstrated to be a powerful tool for the characterization of the amyloid fibril structure [31]. In particular, DUVRR spectroscopy combined with hydrogen-deuterium exchange has been utilized in the structural characterization of the fibril core [27]. A typical protein DUVRR spectrum is dominated by amide bands, which characterize the polypeptide backbone conformation. In addition, the spectrum displays aromatic amino acid bands, which provide information about their local environment [32]. We observed a gradual increase in Raman band intensity with incubation time for C_α_–H (1390 cm^−1^) as well as Amide I and II modes, indicating β-sheet formation. A significant decrease in tyrosine band intensity was also observed, indicating the changes in the local environments of tyrosine residues during protein aggregation (Figure 3, A).

Previous studies of native insulin conformation have indicated that tyrosines have a hydrophilic environment [33], [34]. The decrease in the intensity of aromatic bands in the DUVRR spectrum of insulin fibrils could indicate that tyrosine residues are in a more hydrophobic environment than in the native protein. However, a significant change in the phenylalanine (1000 cm^−1^) band intensity was not observed, indicating that the local environments of phenylalanine residues do not change. These data corroborate our NMR results on the aromatic amino acid environments.

In an unordered protein spectrum, Mikhonin and Asher showed that H/D exchange caused a downshift of the amide II DUVRR band from 1555 to 1450 cm-1 (Amide II’) and the virtual disappearance of the amide III band [35]. Figure 3 B illustrates the corresponding spectral changes in the DUVRR signature of insulin fibrils upon deuteration. The deuteration of mature insulin fibrils resulted in a slight decrease in Amide II band intensity, indicating that the amount of fibril protein available for deuteration was very small. This finding is also corroborated by the relatively small intensity of the Amide II band in the fibril spectrum compared with that of unfolded insulin completely exposed to H/D exchange. The unfolded state of insulin was achieved by dissolution of bovine insulin in D_2_O, pD 1.0 and brief heating at 95°C for several minutes.

Based on our observations, insulin fibrils mainly consist of highly organized cross-β-sheets with high resistance to H/D exchange. According to Asher and coworkers, the position of the Amide III_3_ band corresponds to the Ψ dihedral angle. We observed that insulin fibrils have a single peak centered at 1226 cm^−1^, which, according to Asher’s semi-empirical approach, [35] corresponds to β-sheet conformation characterized by a Ψ dihedral angle of 134.5°.

### MD Simulations of β-sheet-rich Dimer Formation

According to our experimental data, in addition to tertiary changes, dramatic secondary structure changes occurred upon insulin aggregation, although the disulfide bonds remained intact. An obvious question to address is whether the types of structural perturbations that take place in the insulin monomer during fibrillation to satisfy both of these criteria. To demonstrate the possibility of the conversion of the mainly α-helical monomer into the predominantly β-sheet-rich fibril form, we performed all-atom MD simulations in aqueous solution on both monomeric and dimeric forms of insulin. The latter was constructed to represent only the fibrillar form of insulin and the existence of other non-structured oligomeric states was ignored. These simulations provided both the structure and location of each amino acid residue at the atomic level. The accuracy of the simulated structures was validated by comparing them with the available experimental DUVVR and NMR data reported in this study. The structures of the full-length insulin were investigated in the following sequence: monomer → dimer. The most representative structure obtained from the simulation of the monomeric form was used to develop models for the dimeric form. The equilibrated structure of the full-length insulin monomer derived from a 100-ns MD simulation was found to contain mostly α-helical (49.0%) and small β-sheet (3.9%) character (Table 1).

**Table 1:** The α-helix and β-sheet character of simulated insulin monomer and dimer.

Structure	α-helix character	β-sheet character
Monomer	49.0%	3.9%
Dimer	0.0%	43.1%

**Figure 4:**
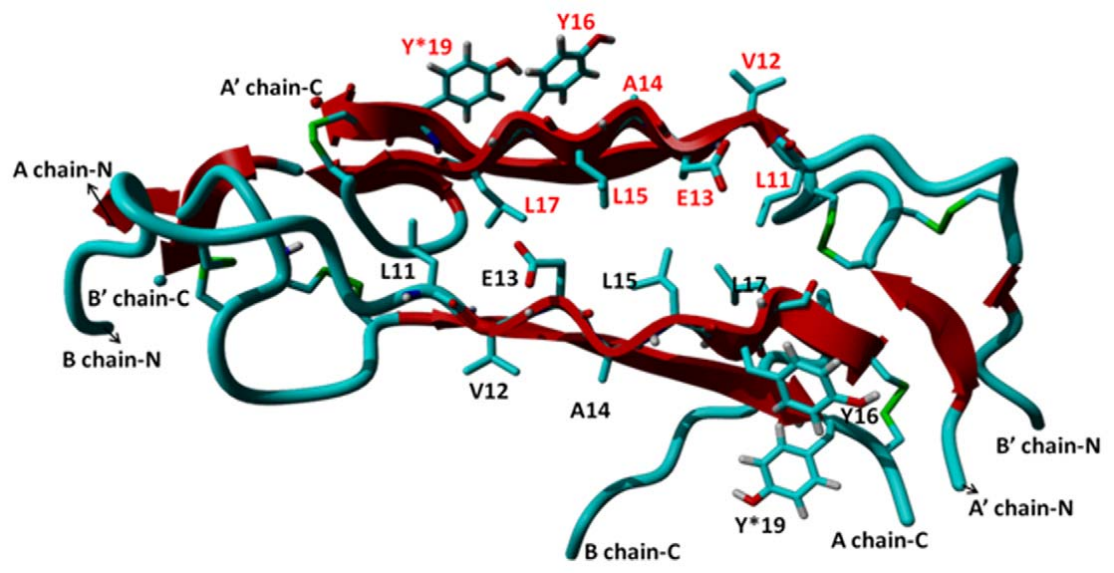
Equilibrated structure of an insulin dimer from 150-ns MD simulations in aqueous solution. The A- and B-chain of the second monomer are labeled À and B̀, respectively. Intact disulfide bridges are shown in green.

This structure is well folded and stabilized by a large number of hydrogen bonds and hydrophobic interactions between A- and B-chains, including their N- and C-termini. In order to form a dimer, the structure must substantially unfold to associate with another monomer (Figure 4). As discussed in the “computational procedure” section, this unfolding is accomplished by applying a constant force on the N- and C-termini of the monomer. During the unconstrained simulations of the dimer, both insulin monomers undergo significant structural changes.

**Figure 5:**
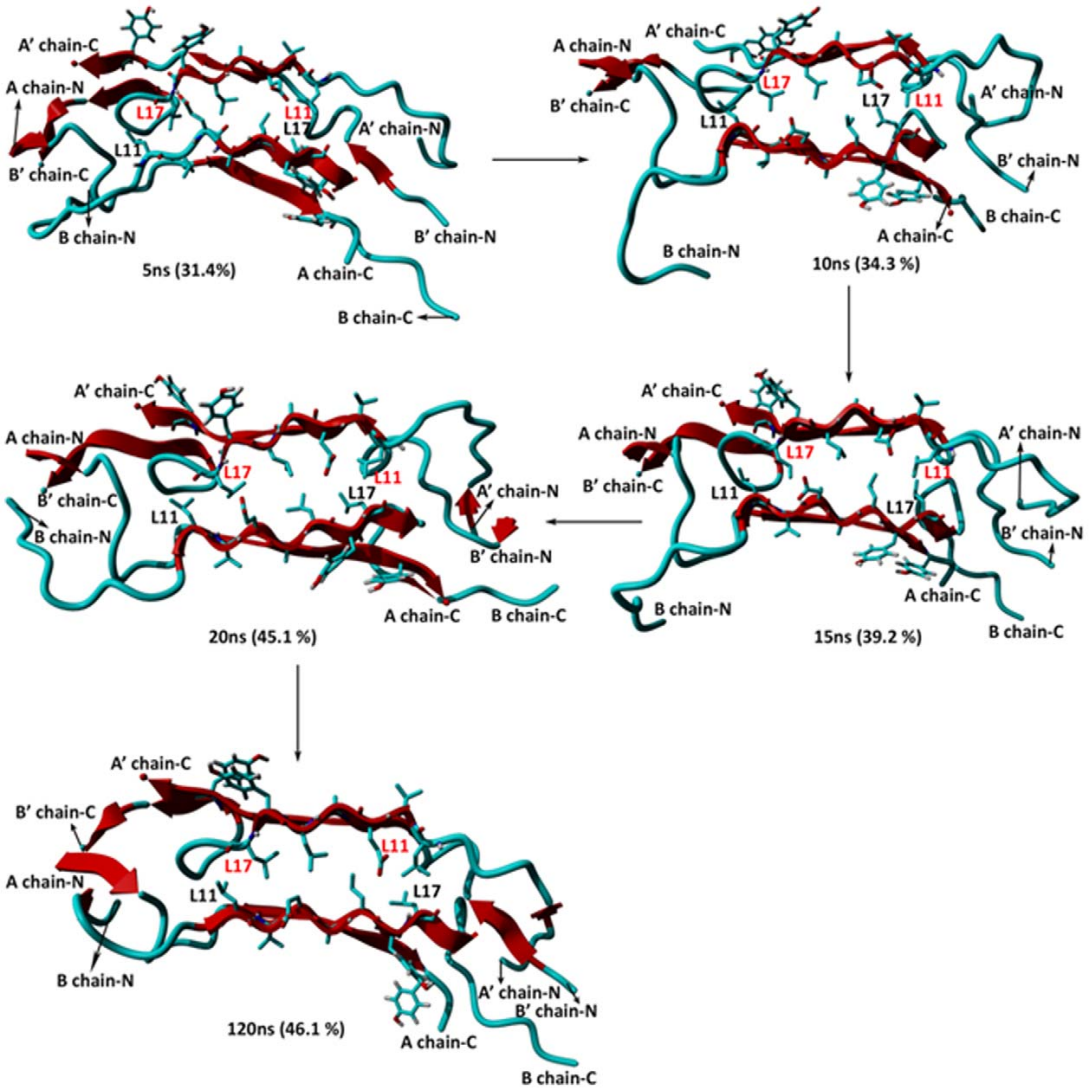
Snapshots of insulin dimers from MD simulations. The β-sheet character of each structure is shown in parentheses.

The time evolution of the 150-ns MD simulation of the dimer shows that there is a gradual increase in the β-sheet character compared with that of the monomer (Table 1). In the first 5 ns of the simulation, the β-sheet content enhanced sharply (3.9 → 31.4%). Subsequently, the following slow increase occurred: 34.3, 39.2, 45.1 and 46.1% at 10, 15, 20 and 120 ns, respectively (Figure 5). The β-sheet character largely remained unchanged in the 120–150-ns time period. By contrast, the α-helix character was significantly reduced from 49.0% to 0.0% (Table 1). Notably, within the dimer, the N- and C-termini of the A- and B-chains of both monomers did not interact with each other. However, in fibrils, these regions most likely associate through β-sheet interactions with other dimers, increasing the content of this secondary structure.

Dimerization occurs through a zipper-like mechanism in which the B10–B18 regions of monomers form two sides of the zipper. The formation of this type of structure has previously been observed in the oligomerization of short fragments of insulin and other amyloidogenic peptides [18], [36]. The information provided by the MD simulations can be combined with the measured H/D exchange data to elucidate the structure of the insulin fibril. In the structure of the zipper derived from simulations, Leu^11^, Leu^15^ and Leu^17^ residues of one monomer interact with their counterparts in the second monomer through hydrophobic interactions. In addition, two tyrosine residues (Tyr^19^ and Tyr^16^ of A and B chains, respectively) interact with each other through π-π interactions. Due to His^10^, Leu^11^, Val^12^, Glu^13^, Ala^14^, Tyr^16^, Leu^17^, Val^18^, Cys^19^, and Gly^20^ residues forming the hydrophobic fibril core, these residues must be protected under H/D exchange. The location and orientation of all these residues in the simulated structure were found to be in excellent agreement with current and previously reported experimental data [18]. In addition, the protection of free Gln^15^, Tyr^19^ and Asn^21^ residues of the A-chain and the Phe^25^-Pro^28^ segment of the B-chain is also in accord with the collected DUVVR and NMR data. Furthermore, as suggested by the experimental data, all three disulfide bonds [Cys^6^(A)-Cys^11^(A), Cys^7^(A)-Cys^7^(B), and Cys^20^(A)-BCys^19^(B)] remained intact in the dimer. Based on these calculations, we confirmed that the tertiary and secondary structures of insulin can dramatically change during oligomerization without breaking disulfide bonds.

Based on these results, we concluded that the dimeric structure of full-length insulin is a good model for elucidating several key structural properties of amyloid fibrils. Dimerization appears to be a critical step in fibrillation. After dimerization, fibrils can grow through the stacking.

**In conclusion**, our new approach of combining NMR and Raman spectroscopy with MD simulations for characterizing amyloid fibrils has provided exclusive knowledge about fibril structure. With single-residue resolution, we determined the amino acid residues that form the fibril core in addition to those that are located in the unordered parts of the fibril. We found that most of the sequence from the B-chain of insulin is highly protected from H/D exchange, including a segment previously described by Eisenberg [18]. However, we did not find any long (not longer than two amino acids) protected segments in the A-chain. Moreover, the A-chain was observed to have the following intriguing order of protection: starting from Cys^11^ to Asn^21^, with alternating protected and unprotected amino acid residues.

We demonstrated that three out of four tyrosine amino acid residues packed into the cross-β-sheet during insulin aggregation, whereas another aromatic amino acid, phenylalanine, remained in the unordered parts. Based on NMR data, we determined that the following B-chain residues are highly protected: Phe^25^, Tyr^26^, and Thr^27^; Lys^29^ and Ala^30^. In addition, we found that both amino acids at the insulin C-termini were unprotected, whereas both amino acids at the N-termini remained highly protonated, packing into the cross-β-core. The location and orientation of these residues and secondary structures of the Phe^25^-Pro^28^ region of the B-chain were supported by structures derived from MD simulations. Furthermore, the structures showed that all three disulfide bonds remained intact in the dimer, which models the fibrillar form of insulin.

Together with the determination of the amino acid sequence that directly participates in the association and fibrillation of insulin dimers, we discovered a unique organization of six insulin cysteine residues, supporting the presence of intact disulfide bonds and their lack of scrambling during insulin aggregation. These results indicate that cysteine residues localized on the fibril surface may play a direct role in free radical formation, which has been previously described for sulfur atoms in proteins [16].

Twenty out of 51 amino acids in the insulin sequence demonstrated complete H/D exchange. We determined that 10 residues are hydrophilic and 10 are hydrophobic (Table S1). Thus, our preliminary estimation of the insulin fibril surface based on NMR data analysis of unprotected amino acids indicates that it is equally hydrophilic (polar) and hydrophobic (nonpolar). The importance of identifying solvent-exposed residues in fibrils is underlined by the fact that the fibrillar surface is one of the major sources of fibrillar toxicity.

## Materials and Methods

### Fibril Formation

Bovine insulin was purchased from Sigma-Aldrich (I5500). Insulin fibrillation was performed by growing insulin (60 mg/ml) in HCl, pH 1.9 at 65°C overnight as previously described [37]. The amyloid fibrils were washed with HCl, pH 1.9 and centrifuged for 30 min at 12,000 g at 25°C. The supernatant was removed and the process was repeated twice. The insulin fibrils were then redispersed in HCl, pH 1.9 and lyophilized. To prepare a protonated sample, 28 mg of lyophilized powder was dissolved in d_6_-DMSO and 0.05% TFA for NMR analysis. To prepare a deuterated sample, 25 mg of lyophilized powder was exposed to D_2_O for 7 days at 20.5°C, followed by lyophilization. Deuterated lyophilized amyloid fibrils were dissolved in d_6_-DMSO and 0.05% TFA for NMR analysis. The final concentrations of the protonated and deuterated amyloid fibrils were 9 mM and 10 mM, respectively.

### NMR Experiments

All samples were placed in a 5-mm NMR tube, and the experiments were conducted on a Bruker AM-500 spectrometer with a *z*-axis gradient cryoprobe. The probe temperature was maintained at 30°C. ^1^H,^1^H-TOCSY and ^1^H,^1^H NOESY spectra were collected with a mixing time of 45 ms and 150 ms, respectively, to optimize magnetization transfer. Spectra were collected using the Watergate pulse sequence for water suppression [38]. All spectra were processed using TOPSPIN 2.1 (Bruker, Inc). In t_1_ and t_2_ dimensions, 4096 and 512 points were collected, respectively. The 2D data sets were apodized by a sine-bell and Fourier transformed. NMR chemical shift assignments were made using CARA software [39].

To date, the bacterial recombinant expression of mature insulin is not possible. Homonuclear NMR is the only method available for assigning protons of insulin. The chemical shift assignments of dispersed insulin amyloid fibrils were assigned based on known assignments of the insulin monomer dissolved in 65% H_2_O, 35% d_3_-acetonitrile, and 0.05% TFA [40]. Because the ^1^H,^1^H-TOCSY spectrum of monomeric insulin was very similar to that of amyloid fibrils, we used the insulin monomer during chemical shift assignment experiments. To match the chemical shifts of insulin under different buffer conditions, 1 mM of insulin monomer was dissolved in H_2_O, 35% d_3_-acetonitrile, and 0.05% TFA, and the solvent was gradually changed to d_6_-DMSO and 0.05% TFA. The changes in the NMR spectra of monomeric insulin were monitored by ^1^H,^1^H-TOCSY.

### Raman Experiments

#### Non-resonance Raman spectroscopy

Insulin fibrils were lyophilized and the resulting insulin protein powder was placed onto alumina foil. A Renishaw inVia confocal Raman spectrometer equipped with a research-grade Leica microscope, 20×long-range objective (numerical aperture of 0.35), and WiRE 2.0 software was used for non-resonance Raman spectroscopy. A 785-nm-wavelength laser was used, and the laser power was reduced to approximately 11.5 mW to avoid sample photo-degradation.

#### Deep UV resonance Raman spectroscopy (DUVRR)

DUVRR spectra (197-nm excitation) were collected using a home-built Raman spectrometer as previously described [41]. A spinning quartz NMR tube with a magnetic stirrer inside was used for sampling. Raman scattering was dispersed and recorded using a homebuilt double monochromator coupled to a liquid-nitrogen-cooled CCD camera (Roper Scientific, Inc.). All reported Raman spectra were an average of at least three independent measurements. GRAMS/AI 7.0 software (Thermo Galactic, Salem, NH) was used for data processing. The application of hydrogen-deuterium exchange combined with DUVRR spectroscopy for structural characterization of the fibril core has been previously described. [42] Samples (1 mL) of fibrils dispersed in water were centrifuged at 14000×*g* for 30 min. The precipitate was subsequently divided into two parts, each of which was washed two times with either D_2_O or H_2_O by subsequent spinning-redispersion. The washed dispersions (in D_2_O and H_2_O) were used for Raman spectroscopic measurements.

### Dynamic Light Scattering (DLS)

Solutions of insulin protein and insulin fibrils disintegrated by DMSO/TFA were analyzed using Dyna Pro Titan DLS (Wyatt Technology Corp.). Acquired data were analyzed using DYNAMICS V6 software (Wyatt Technology Corp.).

### Atomic Force Microscopy (AFM)

Fibril solution was centrifuged at 12,000 g prior to the deposition to remove nonaggregated protein. A gelatinous pellet was diluted with HCl, pH 1.9 or DCl, pD* 1.9 solution with a 1∶400 dilution factor (V/V). A drop of this solution was placed onto freshly cleaved mica in AFM fluid chamber and incubated for 2 min followed by removing of the solution excess. To avoid mica surface drying, 2 ml of distill water were placed on the top of the mica. AFM scanning was performed immediately in tapping mode using MFP-3D™ Bio Asylum Research microscope (Asylum Research, CA, USA) with Olympus TR400PSA tips.

### Computational Methods

Using the GROMACS program [43], [44], all MD simulations were performed utilizing the GROMOS force field GROMOS96 53A5 [45]. In the simulations, the starting structures were placed in a large cubic box (9.0×7.0×7.0 Å^3^) to avoid artificial interactions with their images in the neighboring boxes created by the application of periodic boundary conditions (PBCs). The box was filled with single point charge (SPC) water molecules. The GROMOS96 force field and SPC water model have been successfully employed to explore protein dynamics in several recent studies (cite 1–3 from the letter of response). Some water molecules were replaced with sodium and chloride ions to neutralize the system and to simulate an experimentally used ion concentration of 150 mM. Subsequently, the starting structures were energy-minimized with a steepest descent method that used 3000 steps. The results of these minimizations produced the starting structure for the MD simulations. Subsequently, the simulations were performed with a constant number of particles (N), pressure (P) and temperature (T) (i.e., the NPT ensemble). The SETTLE algorithm [46] was used to constrain the bond lengths and angles of the water molecules, and the LINCS algorithm [47] was used to constrain the bond length of the peptide bond. Long-range electrostatic interactions were calculated by the Particle-Mesh Ewald (PME) method [48]. A constant pressure of 1 bar was applied with a coupling constant of 1.0 ps. Peptides, water molecules and ions were coupled separately to a bath at 300 K with a coupling constant of 0.1 ps. The equation of motion was integrated at each 2-fs time step. Umbrella pulling, as implemented in the GROMACS package, was applied to unfold the monomeric structure. In this method, a harmonic potential is applied between the center of mass of two groups. A standard pulling rate and a force constant of 0.002 nm/ps and 1600 kJ/mol nm^2^, respectively, were used [49]. The tools available in the GROMACS program package and the YASARA program [50] were used for analyzing the trajectories and the simulated structures.

### Computational Modeling

The monomer X-ray structure of the bovine insulin hexamer dimer was crystallized from bovine insulin hexamer (PDB ID: 2ZP6). [51] The insulin monomer was abstracted from the X-ray structure of bovine insulin hexamer (PDB ID: 2ZP6). This structure was simulated in water without any constraints for 100 ns. In the next step, umbrella pulling was applied on the N- and C-termini to unfold this structure. The unfolded structure was then used to prepare a model for the dimer. In the model for the 150-ns simulation, both monomers were oriented to form a zipper-like conformation. The most representative structure obtained from this simulation was further utilized to develop 10-ns MD simulations for the insulin hexamer. In this model, the B6L-B20G and A5Q-A21N fragments of each monomer were truncated.

## Supporting Information

Figure S1 AFM images of insulin fibrils before H/D exchange (a) and two days after incubation in D_2_O, pD* 1.9 at 25°C.
(DOCX)

Figure S2 **DLS data of insulin disintegrated fibrils by DMSO/TFA (A) and insulin protein in HCl, pH 2.0 (B).**
(DOCX)

Figure S3 **The protection degree of each amino acid residue in the insulin monomer.**
(DOCX)

Figure S4 **Backbone conformations of fibrillar and monomer insulins are very similar in the vicinity of disulfide bridges.**
(DOCX)

Table S1 **Hydrophilic (polar) and hydrophobic (nonpolar) amino acids that were completely accessible for H/D exchange.**
(DOCX)

## References

[1] Dobson CM (2003). Protein folding and misfolding.. Nature.

[2] Makarava N, Baskakov IV (2008). The same primary structure of the prion protein yields two distinct self-propagating states.. J Biol Chem.

[3] Goldsbury CS, Wirtz S, Muller SA, Sunderji S, Wicki P (2000). Studies on the in vitro assembly of a beta 1–40: implications for the search for a beta fibril formation inhibitors.. J Struct Biol.

[4] Lindquist SL, Kelly JW (2011). Chemical and biological approaches for adapting proteostasis to ameliorate protein misfolding and aggregation diseases: progress and prognosis.. Cold Spring Harb Perspect Biol.

[5] Dobson CM (1999). Protein misfolding, evolution and disease.. Trends Biochem Sci.

[6] Mossuto MF, Bolognesi B, Guixer B, Dhulesia A, Agostini F (2011). Disulfide bonds reduce the toxicity of the amyloid fibrils formed by an extracellular protein.. Angew Chem Int Ed Engl.

[7] Groenning M, Frokjaer S, Vestergaard B (2009). Formation mechanism of insulin fibrils and structural aspects of the insulin fibrillation process.. Curr Protein Pept Sci.

[8] Jimenez JL, Nettleton EJ, Bouchard M, Robinson CV, Dobson CM (2002). The protofilament structure of insulin amyloid fibrils.. Proc Natl Acad Sci U S A.

[9] Liu C, Sawaya MR, Eisenberg D (2011). beta-microglobulin forms three-dimensional domain-swapped amyloid fibrils with disulfide linkages.. Nat Struct Mol Biol.

[10] Katou H, Kanno T, Hoshino M, Hagihara Y, Tanaka H (2002). The role of disulfide bond in the amyloidogenic state of beta(2)-microglobulin studied by heteronuclear NMR.. Protein Sci.

[11] Kurouski DL, Lednev IK (2011). The impact of protein disulfide bonds on the amyloid fibril morphology.. Int J Biomedical Nanoscience and Nanotechnology.

[12] Chang SG, Choi KD, Jang SH, Shin HC (2003). Role of disulfide bonds in the structure and activity of human insulin.. Mol Cells.

[13] Dische FE, Wernstedt C, Westermark GT, Westermark P, Pepys MB (1988). Insulin as an amyloid-fibril protein at sites of repeated insulin injections in a diabetic patient.. Diabetologia.

[14] Westermark P, Wernstedt C, Wilander E, Hayden DW, O’Brien TD (1987). Amyloid fibrils in human insulinoma and islets of Langerhans of the diabetic cat are derived from a neuropeptide-like protein also present in normal islet cells.. Proc Natl Acad Sci U S A.

[15] Zako T, Sakono M, Hashimoto N, Ihara M, Maeda M (2009). Bovine insulin filaments induced by reducing disulfide bonds show a different morphology, secondary structure, and cell toxicity from intact insulin amyloid fibrils.. Biophys J.

[16] Schoneich C (2002). Redox processes of methionine relevant to beta-amyloid oxidation and Alzheimer’s disease.. Arch Biochem Biophys.

[17] Yip CC, Ottensmeyer P (2003). Three-dimensional structural interactions of insulin and its receptor.. J Biol Chem.

[18] Ivanova MI, Sievers SA, Sawaya MR, Wall JS, Eisenberg D (2009). Molecular basis for insulin fibril assembly.. Proc Natl Acad Sci U S A.

[19] Nayak A, Sorci M, Krueger S, Belfort G (2009). A universal pathway for amyloid nucleus and precursor formation for insulin.. Proteins.

[20] Gibson TJ, Murphy RM (2006). Inhibition of insulin fibrillogenesis with targeted peptides.. Protein Sci.

[21] Nielsen L, Frokjaer S, Brange J, Uversky VN, Fink AL (2001). Probing the mechanism of insulin fibril formation with insulin mutants.. Biochemistry.

[22] Brange J, Andersen L, Laursen ED, Meyn G, Rasmussen E (1997). Toward Understanding Insulin Fibrillation.. Journal of Pharmaceutical Sciences.

[23] Sawaya MR, Sambashivan S, Nelson R, Ivanova MI, Sievers SA (2007). Atomic structures of amyloid cross-beta spines reveal varied steric zippers.. Nature.

[24] Ivanova MI, Thompson MJ, Eisenberg D (2006). A systematic screen of beta(2)-microglobulin and insulin for amyloid-like segments.. Proc Natl Acad Sci U S A.

[25] Hoshino M, Katou H, Hagihara Y, Hasegawa K, Naiki H (2002). Mapping the core of the beta(2)-microglobulin amyloid fibril by H/D exchange.. Nat Struct Biol.

[26] Shashilov VA, Sikirzhytski V, Popova LA, Lednev IK (2010). Quantitative methods for structural characterization of proteins based on deep UV resonance Raman spectroscopy.. Methods.

[27] Xu M, Shashilov V, Lednev IK (2007). Probing the cross-beta core structure of amyloid fibrils by hydrogen-deuterium exchange deep ultraviolet resonance Raman spectroscopy.. J Am Chem Soc.

[28] Cavanagh J, Fairbrother WJ, Palmer AG, Rance M, Skelton NJ (2007). Protein NMR spectroscopy..

[29] Wuthrich K (1986). NMR of proteins and nucleic acids..

[30] Tu AT (1982). Raman Spectroscopy in Biology: Principles and Applications..

[31] Shashilov VA, Lednev IK (2010). Advanced statistical and numerical methods for spectroscopic characterization of protein structural evolution.. Chem Rev.

[32] Lednev IK, Uversky VN, Permyakov EA (2007). Vibrational Spectroscopy: Biological Applications of Ultraviolet Raman Spectroscopy.. Protein Structures, Methods in Protein Structures and Stability Analysis: Nova Science Publishers, Inc.

[33] Whittingham JL, Scott DJ, Chance K, Wilson A, Finch J (2002). Insulin at pH 2: structural analysis of the conditions promoting insulin fibre formation.. J Mol Biol.

[34] Zoete V, Meuwly M, Karplus M (2004). A comparison of the dynamic behavior of monomeric and dimeric insulin shows structural rearrangements in the active monomer.. J Mol Biol.

[35] Mikhonin AV, Bykov SV, Myshakina NS, Asher SA (2006). Peptide secondary structure folding reaction coordinate: correlation between uv raman amide III frequency, Psi Ramachandran angle, and hydrogen bonding.. J Phys Chem B.

[36] Eisenberg D, Nelson R, Sawaya MR, Balbirnie M, Sambashivan S (2006). The Structural Biology of Protein Aggregation Diseases: Fundamental Questions and Some Answers.. Accounts of Chemical Research.

[37] Kurouski D, Lombardi RA, Dukor RK, Lednev IK, Nafie LA (2010). Direct observation and pH control of reversed supramolecular chirality in insulin fibrils by vibrational circular dichroism.. Chem Commun.

[38] Piotto M, Saudek V, Sklenar V (1992). Gradient-tailored excitation for single-quantum NMR spectroscopy of aqueous solutions.. J Biomol NMR.

[39] Masse JE, Keller R, Pervushin K (2006). SideLink: automated side-chain assignment of biopolymers from NMR data by relative-hypothesis-prioritization-based simulated logic.. J Magn Reson.

[40] Kline AD, Justice RM (1990). Complete sequence-specific 1 H NMR assignments for human insulin.. Biochemistry.

[41] Lednev IK, Ermolenkov VV, He W, Xu M (2005). Deep-UV Raman spectrometer tunable between 193 and 205 nm for structural characterization of proteins.. Anal Bioanal Chem.

[42] Xu M, Shashilov V, Ermolenkov VV, Fredriksen L, Zagorevski D, Lednev IK (2007). The first step of hen egg white lysozyme fibrillation, irreversible partial unfolding, is a two-state transition.. Protein Sci.

[43] Berendsen HJC, van der Spoel D, van Drunen D (1995). GROMACS: A message-passing parallel molecular dynamics implementation.. Comput Phys Commun.

[44] Lindahl E, Hess B, van der Spoel D (2001). GROMACS 3.0: a package for molecular simulation and trajectory analysis.. J Mol Model.

[45] Oostenbrink C, Villa A, Mark AE, van Gunsteren, WF (2004). A Biomolecular Force Field Based of the Free Entahlpy of Hydration and Solvation: The GROMOCS Force-Field Parameter Sets 53A5 and 53A6.. J Comput Chem.

[46] Miyamoto S, Kollman PA (1992). SETTLE: An analytical version of the SHAKE and RATTLE algorithms for rigid water models.. J Comput Chem.

[47] Hess B, Bekker H, Berendsen HJC, Fraaije JGEM (1997). LINCS: A linear constraint solver for molecular simulations.. J Comp Chem.

[48] Darden TA, York D, Pedersen L (1993). Particle mesh Ewald: An N•log(N) method for Ewald sums in large systems.. J Chem Phys.

[49] Kim T, Rhee A, Yip CM (2006). Force-Induced Insulin Dimer Dissoaciation: A Molecular Dynamics Study.. J Am Chem Soc.

[50] Krieger E, Vriend G (2002).

[51] Jaimohan SM, Naresh MD, Arumugam V, Mandal AB (2009). Purification, crystallization and preliminary X-ray diffraction studies of parakeet (Psittacula krameri) haemoglobin.. Acta Crystallogr Sect F Struct Biol Cryst Commun.

