# Supplementary material for: Disulfide Bridges Remain Intact while Native Insulin Converts into Amyloid Fibrils
Source: PLoS One. 2012 Jun 1;7(6):e36989. doi: 10.1371/journal.pone.0036989 (PMC3365881; doi:10.1371/journal.pone.0036989)
Supplement: Figure S1 — AFM images of insulin fibrils before H/D exchange (a) and two days after incubation in D2O, pD* 1.9 at 25°C. (DOCX) [file pone.0036989.s001.docx]

Figure S1. AFM images of insulin fibrils before H/D exchange (a) and two days after incubation in D_2_O, pD* 1.9 at 25 °C
