# Supplementary material for: Disulfide Bridges Remain Intact while Native Insulin Converts into Amyloid Fibrils
Source: PLoS One. 2012 Jun 1;7(6):e36989. doi: 10.1371/journal.pone.0036989 (PMC3365881; doi:10.1371/journal.pone.0036989)
Supplement: Figure S2 — DLS data of insulin disintegrated fibrils by DMSO/TFA (A) and insulin protein in HCl, pH 2.0 (B). (DOCX) [file pone.0036989.s002.docx]

Figure S2. DLS data of insulin disintegrated fibrils by DMSO/TFA (A) and insulin protein in HCl, pH 2.0 (B). Insulin monomer, which has a radius of 0.07 nm, takes 99.9% of the solution mass (A). At the same time, insulin protein stays as a dimer (B) with a radius ~2.5 nm, in HCl, pH 2.0 [1].

1. Whittingham JL, Scott DJ, Chance K, Wilson A, Finch J, et al. (2002) Insulin at pH 2: structural analysis of the conditions promoting insulin fibre formation. J Mol Biol 318: 479-490.
