# Supplementary material for: Disulfide Bridges Remain Intact while Native Insulin Converts into Amyloid Fibrils
Source: PLoS One. 2012 Jun 1;7(6):e36989. doi: 10.1371/journal.pone.0036989 (PMC3365881; doi:10.1371/journal.pone.0036989)
Supplement: Figure S3 — The protection degree of each amino acid residue in the insulin monomer. (DOCX) [file pone.0036989.s003.docx]

Figure S3.The protection degree of each amino acid residue in the insulin monomer.
