# Supplementary material for: Disulfide Bridges Remain Intact while Native Insulin Converts into Amyloid Fibrils
Source: PLoS One. 2012 Jun 1;7(6):e36989. doi: 10.1371/journal.pone.0036989 (PMC3365881; doi:10.1371/journal.pone.0036989)
Supplement: Figure S4 — Backbone conformations of fibrillar and monomer insulins are very similar in the vicinity of disulfide bridges. (DOCX) [file pone.0036989.s004.docx]

Figure S4. Backbone conformations of fibrillar and monomer insulins are very similar in the vicinity of disulfide bridges. Overlay between 1H,1H-TOCSY spectra of 1 mM fibryllar(red) and monomer (black) insulins in d6-DMSO and 0.05% TFA at 30 °C. The amino acids located within 4 residues from insulin cysteins are labeled. NMR data were acquired on Bruker Avance 500 MHz NMR spectrometer equipped with the cryoprobe.
