# Supplementary material for: Disulfide Bridges Remain Intact while Native Insulin Converts into Amyloid Fibrils
Source: PLoS One. 2012 Jun 1;7(6):e36989. doi: 10.1371/journal.pone.0036989 (PMC3365881; doi:10.1371/journal.pone.0036989)
Supplement: Table S1 — Hydrophilic (polar) and hydrophobic (nonpolar) amino acids that were completely accessible for H/D exchange. (DOCX) [file pone.0036989.s005.docx]

| hydrophilic (polar) | hydrophobic (nonpolar) |
| --- | --- |
| A-chain | |
| E4 | G1 |
| Q5 | A8 |
| C6, C7, C20 | L17 |
| S9, S12 |  |
| Y14 |  |
| N18 |  |
| B-chain | |
| S9 | F1, F24 |
|  | V2 |
|  | G8, G20, G23 |
|  | P28 |

Table S1. Hydrophilic (polar) and hydrophobic (nonpolar) amino acids that were completely accessible for H/D exchange.
